# A Case Report of an Emergency Hypertensive Episode During Trastuzumab Therapy

**DOI:** 10.7759/cureus.17015

**Published:** 2021-08-09

**Authors:** Adriano Pacheco Mendes, Jerina Nogueira, André Mendes, Joana Cochicho, Isabel Lavadinho

**Affiliations:** 1 Internal Medicine Department, Hospital Dr. José Maria Grande - Unidade Local de Saúde do Norte Alentejano, Portalegre, PRT

**Keywords:** trastuzumab, breast cancer, hypertension, intracerebral hemorrhage, cardio-oncology

## Abstract

Human epidermal growth factor receptor 2 (HER2+) targeted therapies, such as trastuzumab emtansine, has been shown to have a significant positive effect on the outcome of breast cancer. Rarely, this therapy can cause adverse cardiovascular effects such as cardiac dysfunction, arrhythmias and hypertension. We report a case of a 60-year-old woman who presented to the emergency department with an episode of hypertensive emergency. She had been recently diagnosed with hypertension which was found to be poorly controlled. The recent administration of trastuzumab together with this hypertensive episode led us to suspect trastuzumab as the cause for this rise in her blood pressure. With this case we intend to raise awareness of hypertension, a potentially preventable condition, as an adverse effect of human epidermal growth factor receptor 2 targeted therapies such as trastuzumab. Additionally we propose an appropriate and careful management of arterial hypertension among those receiving this first-in-class drug.

## Introduction

Human epidermal growth factor receptor 2 (HER2+) targeted therapies, such as trastuzumab, have improved significantly the management of breast cancer and its outcome [1-2].

Trastuzumab is a life‐extending therapy for breast cancer patients overexpressing HER2+, despite having a known cardiotoxic risk and an increased risk for cardiovascular events [3-5]. As a primary clinical cardiovascular side effect seen with HER2+ targeted therapies, cardiac dysfunction is manifested by an asymptomatic decrease in left ventricular ejection fraction. Less commonly, developing hypertension may be seen as a secondary effect which is not totally understood [6]. If uncontrolled, systemic arterial hypertension can lead to major events such as hypertensive emergencies [7].

## Case presentation

We report the case of a 60-year-old woman admitted to the emergency department following a minor road traffic accident. The patient had a medical history of breast infiltrating ductal carcinoma (HER2+) under trastuzumab monotherapy for seven months and had been submitted to chemotherapy with FEC regimen (5-fluorouracil, epidoxorubicin and cyclophosphamide) four years before trastuzumab initiation. The patient had recently been diagnosed (six weeks before) with hypertension and started treatment with an angiotensin-converting enzyme inhibitor (enalapril 10 mg once daily) together with a calcium channel blocker (amlodipine 5 mg once daily). Despite treatment, it was still poorly controlled.

**Figure 1:**
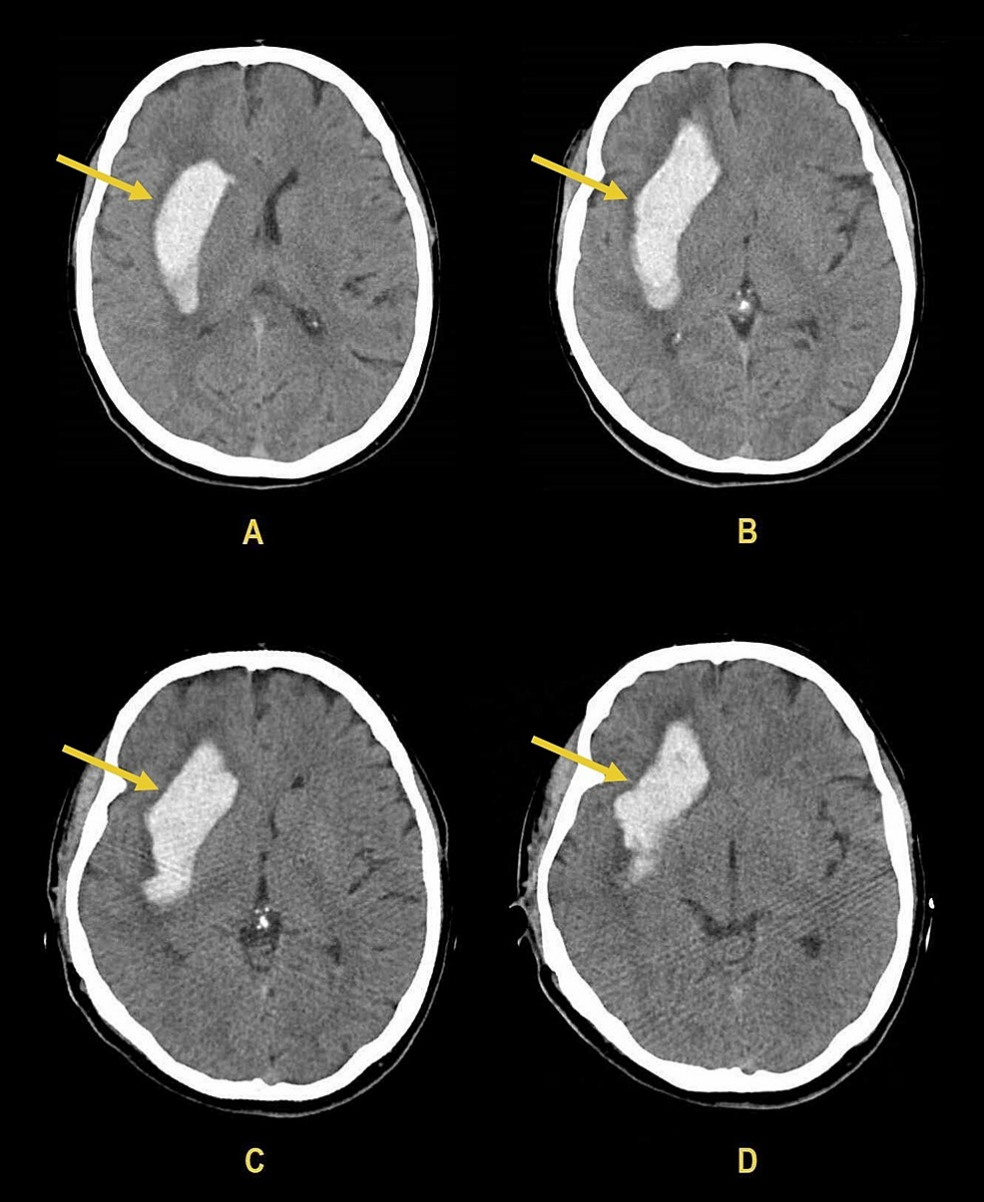
Intracerebral haemorrhage with associated midline shift of 4 mm to the left side on computed tomography scan of the head

On examination in the emergency room, there was no evidence of traumatic lesions in keeping with the paramedics' initial report, the patient was hypertensive (196/97 mmHg), with a Glasgow Coma Scale score of 14 (E4V4M6), following verbal commands but with a new onset left hemiplegia with a National Institutes of Health Stroke Scale (NIHSS) score of 6 (4 points on left arm motor drift and 2 points on left leg motor drift). A non-enhanced computed tomography of the head revealed a right intracerebral haemorrhage of 70 x 40 mm with associated midline shift of 4 mm to the left side (Figure 1).

She was diagnosed with a spontaneous haemorrhagic stroke secondary to uncontrolled blood pressure. Other investigations, such as electrocardiogram and blood analysis, did not show any significant abnormalities. With regards to the transthoracic echocardiogram, it was found to have a segmental wall motion abnormality with a left ventricular mid inferior and basal inferior wall hypokinesia with no compromise of ventricular systolic function. We did not have access to previous echocardiograms for comparison but according to patient’s verbal information, there were no abnormalities on the echocardiography examination performed prior to trastuzumab initiation. Despite information provided by the patient with regards to her medical history, we were unable to safely exclude that the left ventricular regional wall motion abnormality was secondary to the previous chemotherapy regimen or due to a different etiology.

Even though the patient presented with an intracerebral haemorrhage which could lead to a poor outcome, recovery was favourable with minor neurological deficits (NIHSS score of 1 on left arm motor drift) after 10 days of hospitalization. Short follow-up appointment with her oncologist was set up and antihypertensive therapy optimized before discharge (enalapril 20 mg once daily, amlodipine 10 mg once daily and indapamide 2.5 mg once daily).

## Discussion

As medicine takes further steps towards the development of new treatment strategies, more women are surviving and living with breast cancer. Despite the significant improvements in outcomes for women with HER2-positive tumours, breast cancer patients are regarded as a population with high cardiovascular risk [8]. This risk is higher when associated with trastuzumab treatment due to the known adverse effects which increases incidence of cardiovascular conditions such as left ventricular dysfunction, arrhythmias, hypertension and cardiomyopathy [4,9].

Systemic arterial hypertension is described as an uncommon adverse effect [7,10]. Although it could be coincidental, according to the patient’s past medical history, there was a strong temporal association between the onset of arterial hypertension and the timing of initiation of trastuzumab, which she had been taking for the previous seven months, suggesting that it was likely related to trastuzumab exposure. No other evidence pointed to an alternative cause of secondary hypertension.

Identifying risk factors associated with trastuzumab-induced cardiotoxicity allows more targeted and intensive screening for at-risk patients. Since hypertension is a potentially preventable condition during breast cancer treatment and one of the most consistent predictors of trastuzumab-mediated cardiotoxicity, a more regular routine cardiac monitoring is desirable. In addition, surveillance and management of hypertension and other cardiovascular risk factors should be routinely performed during trastuzumab treatment in order to avoid events like hypertensive emergencies and their complications [9,11].

## Conclusions

Given the adverse outcomes associated with uncontrolled hypertension and the prospect of increasing use of trastuzumab for patients with metastatic breast cancer, we attempt to raise awareness to hypertension, a potentially preventable risk factor, as a possible uncommon adverse effect of humanized anti-HER2 positive monoclonal antibodies such as trastuzumab. We also intend to propose an appropriate and careful management of any secondary arterial hypertension incidence among those receiving this first-in-class drug, which continues to have a favourable benefit-risk ratio.
